# Complete genome sequence of the phthalate-degrading bacterium *Gordonia sihwensis* RL-BY03, isolated from mangrove sediment

**DOI:** 10.1128/mra.00777-26

**Published:** 2026-07-27

**Authors:** Yuxuan Zhang, Qiqi Lin, Hanqiao Hu, Lei Ren, Yang Jia, Yanyan Wang

**Affiliations:** 1 College of Coastal Agricultural Sciences, Guangdong Ocean University 74780, Zhanjiang, China; 2 College of Life and Environmental Science, Wenzhou University 26495, Wenzhou, China; Montana State University, Bozeman, Montana, USA

**Keywords:** *Gordonia sihwensis*, mangrove sediment, marine bacterium, complete genome sequence, bioremediation

## Abstract

*Gordonia sihwensis* RL-BY03 is a marine bacterium isolated from intertidal mangrove sediment in Zhanjiang Bay, China, capable of degrading phthalic acid esters (PAEs) as the sole carbon source. Here, we report its complete genome sequence, which provides genetic insights into PAE biodegradation in coastal environments.

## ANNOUNCEMENT

As the major class of additives, phthalic acid esters (PAEs), also known as the most widely used plasticizers, are found in all oceans (1, 2). PAEs are pervasive environmental pollutants with endocrine-disrupting effects (3, 4). Microbial degradation represents a promising remediation strategy, yet genomic resources for marine PAE degraders remain limited (5). Numerous PAE-degrading microorganisms have been isolated and identified, including species from the genera of *Gordonia*, *Mycobacterium*, *Pseudomonas*, *Cupriavidus*, and *Rhodococcus* (6, 7). Strain RL-BY03 was isolated from intertidal mangrove sediment (21.148918°N, 110.443287°E) in Zhanjiang Bay, China, in May 2023, using mineral salt medium (MSM) supplemented with di(2-ethylhexyl) phthalate (DEHP) as the sole carbon source (8). Based on 16S rRNA gene sequence analysis, the strain showed 99.8% similarity to *Gordonia sihwensis* DSM 45487^T^, suggesting its preliminary assignment to the species *G. sihwensis*. The strain was routinely cultured in lysogeny broth (LB) medium (10 g/L tryptone, 5 g/L yeast extract, 25 g/L NaCl, at pH 7.0) at 30°C with shaking (180 rpm).

Strain RL-BY03 was grown in LB medium at 30°C for 24  h prior to DNA extraction. High-molecular-weight genomic DNA was isolated using the TaKaRa MiniBEST Bacterial Genomic DNA Extraction Kit Ver. 3.0. DNA quality was assessed using a NanoDrop spectrophotometer, Qubit fluorometer, and 0.35% agarose gel electrophoresis. Library preparation was performed according to the standard PacBio SMRTbell protocol. Briefly, high-molecular-weight genomic DNA was mechanically sheared using a g-TUBE, followed by DNA damage repair and end repair. Hairpin adapters were ligated to DNA fragments, and exonuclease digestion removed unligated fragments. Target fragments were enriched using the BluePippin system to construct the final SMRTbell library. Sequencing was performed on a PacBio Sequel II platform in the HiFi mode.

Default parameters were used for all software unless otherwise specified. Raw PacBio HiFi circular consensus sequencing (CCS) reads were generated with the official PacBio CCS tool. Adapters were removed, and reads shorter than 2,000 bp or of low quality were filtered, resulting in 92,410 clean reads with a total of 1,024,548,734 bp, an average length of 11,086 bp, an *N*_50_ length of 20,258 bp, and a maximum read length of 289,822 bp. The filtered long reads were *de novo-*assembled using Canu v2.2 (9) with default parameters for bacterial genomes. The assembly pipeline automatically identified overlapping contig ends, removed redundant sequences, and confirmed the circular topology of both the chromosome and plasmid. The completed genome was not rotated to a specific base; the final coordinates were retained as produced by the assembly software without further manipulation.

The final assembly comprises a single circular chromosome (4,255,619 bp) and one circular plasmid (102,746 bp), with an overall G + C content of 68.03% and ~473× sequencing coverage. The plasmid was identified based on its circular assembly structure, the presence of plasmid-specific replication and partition genes, and the absence of significant homology to the main chromosome. The assembly encodes 4,026 predicted protein-coding sequences (CDSs), 51 tRNA genes, and 12 rRNA genes (four copies each of 5S, 16S, and 23S). For species identification, the 16S rRNA gene was amplified using primers 27F (5′-AGAGTTTGATCCTGGCTCAG-3′) and 1492R (5′-GGTTACCTTGTTACGACTT-3′). The PCR product was Sanger-sequenced, and the sequence was compared against the NCBI 16S rRNA database using BLASTn. The closest match was *Gordonia sihwensis* DSM 45487ᵀ with 99.8% sequence identity, with the accession number NR_117031.1.

**Fig 1:**
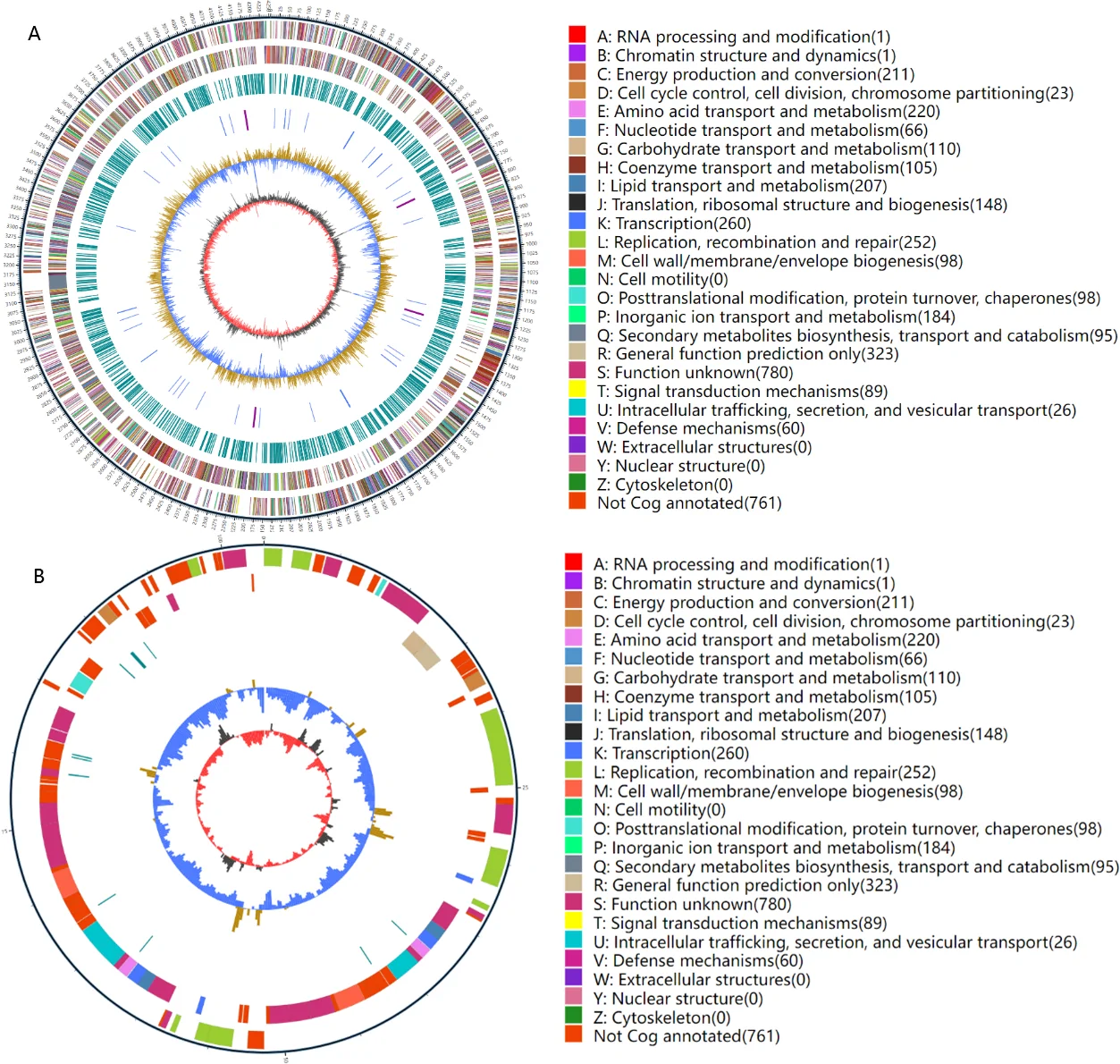
Circular representation of the *Gordonia sihwensis* RL-BY03 chromosome (**A**) and plasmid (**B**). Rings show (from outside to inside) the following: CDSs on forward and reverse strands (colored by COG category), repetitive sequences, RNA genes (tRNAs in blue and rRNAs in purple), GC content, and GC skew.

Functional annotation was performed using the NCBI Prokaryotic Genome Annotation Pipeline (PGAP) v6.5, revealing genetic determinants potentially involved in PAE catabolism, consistent with the strain’s degradative phenotype (10). A circular representation of the genome is shown in Fig. 1.

Full details of all bioinformatics software—including versions, citations, and parameters (with the statement: “Default parameters were used unless otherwise specified”)—as well as methods for genome circularization, plasmid validation, and polishing are available in Figshare under DOI: https://doi.org/10.6084/m9.figshare.31856740 (11).

## Data Availability

This complete genome has been deposited in GenBank under accession numbers CP121248 (chromosome) and CP121249 (plasmid). The raw sequencing reads are available in the Sequence Read Archive (SRA) under BioProject PRJNA947740 and BioSample SAMN33865699. The strain is preserved in the China General Microbiological Culture Collection Center (CGMCC).
